# Prevalence and Risk Factors of Overweight and Obesity among Adolescents and Their Parents in Central Greece (FETA Project)

**DOI:** 10.3390/ijerph13010083

**Published:** 2015-12-25

**Authors:** Anna Patsopoulou, Zoi Tsimtsiou, Antonios Katsioulis, George Rachiotis, Eleni Malissiova, Christos Hadjichristodoulou

**Affiliations:** 1 Department of Hygiene and Epidemiology, Medical Faculty, School of Health Science, University of Thessaly, Larissa 41222, Greece; zoitsimtsiou@yahoo.gr (Z.T.); akatsioul@med.uth.gr (A.K.); grachiotis@gmail.com (G.R.); xhatzi@med.uth.gr (C.H.); 2 Food Technology Department, Technological Educational Institute of Thessaly, Karditsa 43100, Greece; emalisiov@med.uth.gr

**Keywords:** obesity, overweight, adolescents, body mass index, Greece, parental style

## Abstract

The increasing obesity trend in adolescence is a public health concern. The initial phase of Feeding Exercise Trial in Adolescents (FETA) aimed in investigating the prevalence of overweight and obesity in adolescents and their parents and in identifying associated factors among parents’ and adolescents’ demographics, eating habits, and parental style. The sample consisted of 816 adolescents, aged 12–18 years old, and their parents from 17 middle and high schools in Larissa, central Greece. During school visits, anthropometric measurements were performed along with examination of blood pressure. The students completed the study tool that comprised of demographics and the modified versions of Parental Authority Questionnaire (PAQ), the Parent-Initiated Motivational Climate Questionnaire-2 (PIMCQ-2) and the Family Eating and Activity Habits Questionnaire (FEAHQ). Their parents completed a questionnaire with demographics, anthropometrics and FEAHQ. Normal Body Mass Index was found in 75.2% of the adolescents, 2.6% of the adolescents were underweight, 18% overweight and 4.2% obese. Regarding the parents, 76.3% of the fathers and 39.2% of the mothers were overweight or obese. The logistic regression analysis revealed that, overweight or obesity in adolescence was associated with gender (boy), maternal overweight or obesity, lower maternal educational level, eating without feeling hungry, eating in rooms other than kitchen and having a father that motivates by worrying about failing. A significant proportion of adolescents and their parents are overweight or obese. Future interventions should focus both on the parents and children, taking into account the role of parental authority style, in preventing adolescents’ obesity.

## 1. Introduction

Obesity in early years of life appears to be an important predictor of adult obesity and morbidity [1], while it is currently considered the most prevalent nutritional disease of children and adolescents in developed countries [2]. Previous studies in Greece have showed that the prevalence of childhood overweight and obesity is high, being in line with the reports of other southern European countries [3,4], while a recent review presented a tendency for weight increase in children in the last 30 years [5].

Gender [4,6,7], parental weight [7], parental educational level [8], have been among the factors that were associated with obesity in adolescence in previous studies, often with controversial findings. Although in the vulnerable period of adolescence, parents are expected to play a significant role in the development of their children’s attitudes and behaviours, there is a scarcity of studies on the influence of parental style in dietary habits of the children. Parental style is a concept introduced by Baumrind [9], who described three distinct prototypes of parental authority: permissiveness, authoritarianism and authoritativeness. Permissive parents tend to make fewer demands on their children, allowing them to regulate their own activities [9]. Authoritarian parents tend to be very strict and rigid by setting rules for acceptable behaviour. Authoritative parenting style is a combination between the two extremes [9]. Apart from the parents’ authority style, no research has been conducted on the possible influence of the parent-initiated motivational patterns on children’s weight, although previous studies have shown their association with children’s behaviour in athletic activities [10]. Therefore, although parental behaviour is expected to have an impact on adolescents’ eating behaviours, this association has received little research attention [11].

Although several studies have been conducted on the prevalence of overweight and obesity among children in Greece, only a few have investigated overweight and obesity in both adolescents and their parents [12,13,14,15].

The initial phase of FETA project, presented in this paper, is a cross-sectional, school-based study that aimed in investigating the prevalence of overweight and obesity in adolescents (12–18 years old) and their parents. Additionally, it aimed in identifying factors associated with overweight and obesity in adolescence, including apart from parents’ and students’ demographics and eating habits, parental style and perceived motivational climate of parents.

## 2. Method

### 2.1. Study Design

All public middle and high schools (26 in the total) in the city of Larissa in Greece were informed about the purposes of the study. Seventeen secondary and high schools took part in the study with a total of 2648 students, aged from 12 to 18 years old. Secondary education in Greece is compulsory until the age of fifteen, including three years of middle school (from 12 to 15 years old), that can be followed by three more years in high school.

### 2.2. Procedure

During the scheduled school visits, the students that had returned signed informed consents underwent anthropometric measurements, including body weight, height, waist circumference, and also systolic and diastolic pressure and heart rate measurement by a single trained nurse, member of the research team, that followed a standardized procedure. The students were asked to complete anonymously and voluntarily the study that comprised of demographics, medical history (e.g., smoking, diabetes mellitus, *etc*.) and the modified versions of Parental Authority Questionnaire (PAQ) [16], of Parent-Initiated Motivational Climate Questionnaire-2 (PIMCQ-2) [17] and of the Family Eating and Activity Habits Questionnaire (FEAHQ) [18]. Their parents were sent to complete anonymously a questionnaire, which comprised of demographics, their height and weight, their child's medical history and the Family Eating and Activity Habits Questionnaire, while they were given a seven-day period to return the questionnaires. Data collection took place forth months of the school year, from September to January.

### 2.3. Outcome Measures

#### 2.3.1. Students’ Measurements during School Visits

Students were weighed on a digital scale twice and the average was recorded. Participants removed shoes and jackets before height and weight were measured. Weight was measured using Tanita HD646 scales (Tanita Corporation of America Inc., Illinois, IL, USA) to 0.1 kg. Height was measured to 0.1 cm, using the stretch stature method and PE87 portable stadiometers (Mentone Educational Centre, Victoria, Australia). Participants’ height was measured using a metric measuring tape affixed on the wall. Non-extensible steel tapes were used to assess waist circumference, which was measured at the level of the mid point between the lower costal border and the iliac crest. All the anthropometric measures were conducted using the International Society for the Advancement of Kin anthropometry procedures [19]. Body Mass Index (BMI) was determined according to the following formula: BMI = (weight/height^2^). We used the cut off points for the BMI in childhood presented by Cole *et al.* in order to allow international comparisons with our findings in the prevalence of overweight and obesity [20]. Systolic and diastolic blood pressure and heart rate were measured using an automated blood pressure monitor under standardised procedures. 

#### 2.3.2. Parental BMI Calculation

Self-reported parents’ weight and height was used to calculate parental BMI and categorize their weight status according to the Centers for Disease Control and Prevention recommended cut off points for overweight (BMI > 25) and obesity (BMI > 30) [21].

#### 2.3.3. Family Eating and Activity Habits

The modified version of the FEAHQ administered to parents and adolescents, is divided into four subscales: activity level (four items), stimulus exposure (eight items), eating related to hunger (four items), and eating style (thirteen items) [18]. Scores were calculated separately for each member of the family. The questionnaire underwent bilingual translation and cultural adaptation, while internal consistency was estimated with a Cronbach alpha coefficient of 0.51.

#### 2.3.4. Parental Style

The PAQ comprises of 30 items for each parent, presented in a 5-point Likert scale (ranging from 1 = completely disagree to 5 = completely agree) [16]. Ten items describe parental authority behaviour examples, ten items describe parental authoritarian behaviour and ten items are about parental permissiveness. PAQ’s score could range from 10 to 50, with higher scores reflecting more authoritative, authoritarian or permissive parental behavior.

The PIMCQ-2 was used to assess children’s perceptions of the parent-initiated motivational climate. PIMCQ-2 is a 36-item questionnaire (18 items for each parent), which provides scores on three subscales (6 items for each one). The Learning and Enjoyment-Emphasis subscale reflects a mastery orientation, while the Success-without-Effort and Worry-Conducive Behaviours subscales reflect ego orientation [17]. The items can be answered in a 5-point Likert scale (ranging from 1 = completely disagree to 5 = completely agree), while total scores for each subscale can range from 1 to 5, with higher scores indicating a higher agreement with motivation based on the enjoyment of learning, on success without effort or on worry of failure accordingly.

Both questionnaires underwent bilingual translation and cultural adaptation in Greek. Internal consistency was estimated using the Cronbach alpha coefficient. Its values for each of the six PAQ scales were found between 0.514 and 0.836, while for PIMCQ-2 ranged from 0.730 to 0.843.

### 2.4. Ethical Considerations

The Medical School of University of Thessaly and Ministry of Education and Religious Affairs, Sport and Culture has approved the study protocol. Parents were informed about the purposes of the project and were asked to provide written informed consent for participation.

### 2.5. Data Analysis

Quantitative variables are presented as mean with standard deviation, minimum and maximum value or as median with interquartile range (IQR). Qualitative variables are presented as frequencies with percentages. Independent-samples t-test (for normally distributed data) or Mann-Whitney test (for non-normally distributed data) was conducted to investigate associations between binary variables and quantitative variables. Normality of quantitative data was checked using Kolmogorov-Smirnov test.

In univariable analysis, Chi-square test was applied to identify relationships between categorical factors and BMI categories calculating the relative risks (RR) and the corresponding 95% confidence intervals (95% CIs). Chi-square test for trend was used to explore any associations between ordinal factors and overweight-obese adolescents. Logistic regression analysis was performed using the backward conditional method to identify factors associated with obesity-overweight. Variables with p-value less than 0.1 in univariable analysis were included in the logistic regression analysis.

Cronbach’s alpha coefficient used to estimate the internal consistency reliability for the modified versions of the questionnaires PAQ, PIMCQ-2 and FEAHQ. ROC curve analyses were conducted to determine cut-off values of questionnaires’ scores for obesity/overweight. The level of statistical significance was set at *p* value 0.05. Data were analyzed using Epi-Info (version 3.5.3, CDC, Atlanta, GA, USA) and SPSS 19.0 (IBM SPSS Inc., Armonk, NY, USA).

## 3. Results

### 3.1. Descriptive Characteristics and Anthropometric Measurements of Participants

Out of 2648 students who were invited to participate, 1012 parents signed the informed consent (initial response rate 38.2%), expressing interest to participate, while 816 (80.6% of those with signed informed consents) were present during the school visits and participated in the study.

The descriptive characteristics and the anthropometric measurements of the 816 adolescents who participated, as well as statistical significant differences in their means between boys and girls are presented in Table 1. Statistically significant differences were observed in all measurements with boys having higher mean values than girls, except heart rate.

**Table 1:** Descriptive characteristics of participants

Variables	Boys	Girls	*p*-Value
	Mean ± SD (Min–Max)	Mean ± SD (Min–Max)	
***N***	360	456	
**Age (year)**	13.49 ± 1.36 (12–17.5)	13.81 ± 1.51 (11.5–18)	**0.002**
**Weight (Kg)**	58.52 ± 14.06 (30.6–99)	53.19 ± 8.88 (32.8.6–82.6)	**<0.001**
**Height (cm)**	164.45 ± 11.1 (137–188)	160.75 ± 6.69 (141–181)	**<0.001**
**BMI (Kg/m^2^)**	21.44 ± 3.85 (14.02–40.89)	20.52 ± 2.74 (14.98–31.05)	**<0.001**
**Waist Circumference (WC) (cm)**	77.65 ± 10.28 (52–115)	71.85 ± 8.55 (53–137)	**<0.001**
**Pulse (BPM)**	87.18 ± 15.18 (55–141)	91.46 ± 16.19 (50–148)	**<0.001**
**Systolic Blood Pressure (mmHg)**	128.71 ± 15.21 (93–166)	125.43 ± 14.07 (85–167)	**0.002**
**Diastolic Blood Pressure (mmHg)**	75.34 ± 9.67 (41–99)	75.15 ± 8.36 (52–99)	0.769

The families who returned the study’s instrument were 451 (451 mothers and 442 fathers). In terms of educational level for the fathers, 19.5% went to secondary school, 47% to high school and 33.5% went to university. For the mothers, the percentages were 13.5%, 48.1% and 38.4%, accordingly. Mothers had a mean age of 41.5 ± 4.2 years (min = 31, max = 54) and mean BMI 24.7 ± 3.8 Kgr/m^2^ (min = 17.6, max = 39.2), while in the fathers mean age was 46 ± 4.8 years (min = 35, max = 64) and the BMI 27.6 ± 3.7 Kgr/m^2^ (min = 20.5, max = 55.4).

BMI categories of both parents and adolescents are shown in Table 2. Regarding the parents, fathers were found overweight or obese in statistical significantly higher percentages compared to mothers (76.3% *vs*. 39.2%, *p* < 0.001). Respectively for the adolescents, the boys were found in higher percentages overweight or obese compared to their girls classmates (34.8% *vs*. 20.8%, *p* < 0.001).

**Table 2:** BMI categories of parents and children’s.

BMI Categories	Gender **			
	Fathers % (*n/N*)	Mothers % (*n/N*)	Boys % (*n/N*)	Girls % (*n*/*N*)
Underweight	0% (0/442)	1.3% (6/451)	1.9% (3/158)	6.5% (19/293)
Normal weight	23.8% (105/442)	59.4% (268/451)	63.3% (100/158)	72.7% (213/293)
Overweight	55% (243/442)	29% (131/451)	25.9% (41/158)	18.8% (55/293)
Obesity	21.3% (94/442)	10.2% (46/451)	8.9% (14/158)	2% (6/293)

** *p* < 0.001.

### 3.2. Parenting Style

The most frequent parenting style that fathers and mothers rated among the three subscales of parenting styles was authoritative (mean = 35.89 ± standard deviation = 6.12 & mean = 37.49 ± standard deviation = 5.37 respectively). Authoritative parents tend to be located between the edges of the two other styles mentioned above. They are characterized by demand and responsibility. They believe in and acknowledge their rights and duties as parents, and accordingly they supervise and set clear and consistent demands, according to which they expect their children to act. These parents are assertive, yet not invasive or binding, due to their recognition of their child’s uniqueness and will. Their approach is typified by support, warmth, flexibility, abilities to understand and communicate with their children, implementing reason and a “give and take” verbal negotiation instead of punishment and expectations for obedience. These parents are aware to the possibility that they are apt to mistakes in their lives and that they are not a single and definite authority in their children’s lives.

Boys had higher scores in mother’s authoritarianism (median: 25; IQR: 20–29; *p* = 0.043), father’s authoritarianism (median: 26; IQR: 22–32; *p* = 0.010) and mother’s anxiety (median: 2.8; IQR: 2–3.4; *p* = 0.012), while girls scored higher in mother’s learning (median: 4.33; IQR: 3.89–4.67; *p* = 0.037) and father’s learning (median: 4.33; IQR: 3.89–4.67; *p* = 0.024).

### 3.3. Predictors of Being Overweight/Obese in Adolescence

Table 3 presents the results of univariate analysis. Gender (RR = 1.67; 95% CI: 1.23–2.28; *p* < 0.001), BMI group for mother (overweight mothers: RR = 1.78; 95% CI: 1.28–2.49; *p* < 0.001; obese mothers: RR = 1.76; 95% CI: 1.11–2.80; *p* = 0.022), educational level of the mother (secondary school: RR = 2.29; 95% CI: 1.48–3.55; *p* < 0.001; high school: RR = 1.54; 95% CI: 1.05–2.27; *p* = 0.024), having the habit to eat although not hungry (RR = 2.56; 95% CI: 1.89–3.48; *p* < 0.001), eating in rooms other than kitchen (RR = 1.69; 95% CI: 1.14–2.49; *p* = 0.007), and for perceiving that their fathers’ motivation was based on worrying about failure (RR = 1.54; 95% CI: 1.13–2.10; *p* = 0.006) were significantly associated with overweight and obesity in adolescents. It should be noted that the education level of the adolescents was not found to be statistically associated with overweight and obesity, and for this reason it was not further included in the logistic regression analysis.

**Table 3:** Univariate analysis for adolescent’s obesity.

Variables	Categories	Adolescent’s Obesity (Obese/Overweight)		RR	95% CI	*p*-Value
		Frequency	%			
Gender	Boys	55/158	34.8	1.67	1.23–2.28	0.001
	Girls	61/293	20.8	reference		
BMI Groups for Mother	Obesity	16/46	34.8	1.76	1.11–2.80	0.022
	Overweight	46/131	35.1	1.78	1.28–2.49	0.001
	Normal weight	54/274	19.7	reference		
BMI Groups for Father	Obesity	32/94	34.0	1.49	0.95–2.34	0.080
	Overweight	57/243	23.5	1.03	0.68–1.56	0.903
	Normal weight	24/105	22.9	reference		
Educational level for Mother	Secondary School	25/61	41.0	2.29	1.48–3.55	<0.001
	High School	60/217	27.6	1.54	1.05–2.27	0.024
	University	31/173	17.9	reference		
When your child asks to eat does he/she claim to be hungry?	No	31/57	54.4	2.56	1.89–3.48	<0.001
	Yes	83/391	21.2	reference		
How often do you eat in the Living room and in the bedroom	Often/Always	37/132	28.0	1.47	0.97–2.12	0.065
	Sometimes	46/143	32.2	1.69	1.14–2.49	0.007
	Never/Almost never	33/173	19.1	reference		
PIMCQ-2 score of Father (Worry)	2.7+	58/177	32.8	1.54	1.13–2.10	0.006
	<2.7	58/273	21.2	reference		

**RR**, risk ratio; **CI**, confidence interval.

According to the multivariate analysis (Table 4), increased possibility to develop overweight/obesity was associated with being a boy, (OR = 2.01; 95% CI: 1.23–3.27; *p* = 0.005), having an overweight or obese mother, (OR = 2.70; 95% CI: 1.61–4.54; *p* < 0.001), having a mother with lower educational level (OR = 3.39; 95% CI: 1.61–7.12; *p* = 0.001), having the habit to eat although not hungry (OR = 4.37; 95% CI: 2.26–8.46; *p* < 0.001), eating in rooms other than kitchen (OR = 2.09; 95% CI: 1.17–3.75; *p* = 0.013) and for perceiving that their fathers’ motivation was based on worrying about failure (OR = 1.72; 95% CI: 1.06–2.78; *p* = 0.028). We didn’t find significant differences for the factor of father’s overweight/obesity in the multivariate model.

**Table 4:** Predictors of overweight-obese adolescents and their parents in multivariate analysis.

Obese-Overweight *vs*. Normal-Underweight		OR	95% CI	*p*-Value
Age		0.88	(0.73–1.05)	0.150
Gender	Boys	2.01	(1.23–3.27)	0.005
	Girls	reference		
BMI Groups for Mother	Obesity	1.68	(0.74–3.78)	0.214
	Overweight	2.70	(1.61–4.54)	<0.001
	Normal weight	reference		
Educational level for Mother	Secondary School	3.39	(1.61–7.12)	0.001
	High School	1.58	(0.92–2.70)	0.097
	University	reference		
When your child asks to eat. Does he/she claim to be hungry?	No	4.37	(2.26–8.46)	<0.001
	Yes	reference		
How often do you eat in the following rooms? (bedroom, living room)	Often/always	1.82	(1.00–3.32)	0.052
	Sometimes	2.09	(1.17–3.75)	0.013
	Never/Almost never	reference		
PIMCQ-2 score of Father (Worry)	2.7+	1.72	(1.06–2.78)	0.028
	<2.7	reference		

**OR**, odds ratio; **CI**, confidence interval.

## 4. Discussion

In our study, almost one fourth (22.2%) of the adolescents were found overweight or obese, while this percentage was doubled for their mothers (39.2%) and exceeded the three fourths of their fathers (76.3%). Being overweight or obese in adolescence, according to the logistic regression model, was associated with being a boy, having an overweight or obese mother, having a mother with lower educational level, eating without feeling hungry, eating in rooms other than kitchen and having a father that uses the worry about failing for motivation. The prevalence of overweight and obesity in adolescence found in our study is in line with findings reported in earlier Greek and European studies, being at lower edge of the reported ranges [4,6,7,22]. Moreover, the fact that male gender is associated with an increased possibility to develop obesity is in line with numerous previous studies, not only in adolescence, but also in early adult life [4,7,13]. This gender-associated difference has been previously attributed to the fact that young girls’ activity and nutrition is affected by gender norms and feminine ideals through complex negotiations, perceptions, body-centered discourse, and societal influences [23].

Maternal weight and educational status were found predictive of obesity in adolescents, indicating that children tent to “follow in mother’s footsteps”. Our findings are in line with previous research in European countries, suggesting the strong association between lower parental education and obesity [8,24], although there is only one American study presenting the reverse results [25]. However, it is interesting that in an Italian study both parents’ lower educational level is a predictor [26], in an Icelandic mainly fathers’ level [27], while in other Greek, Turkish, Portuguese studies, including ours, only maternal level is identified as a factor associated with increased BMI in their children [7,24,28]. These differences probably reflect the cultural differences in the studied populations and the impact of the roles that parents may have in different societies, implying therefore the central role that mothers have in the Greek family. Similarly, parental obesity has been repeatedly found associated with increased BMI in children [7,24], while in a number of studies it was emphasized that especially maternal obesity is a predicting factor [28]. The strong relationship between children’s and parental BMI is expected to be related to a combination of genetic and lifestyle factors, with the latter being a significant modifiable predictor. Specifically, the maternal role seems to be central in implementing a healthier lifestyle in the family, preventing obesity. Within this context, eating mainly in the kitchen and eating when they feel hungry are among the rules that the parents- especially mothers- are expected to impose in their family, with beneficial results in weight for both children and adults [29].

Adolescents perceiving that their fathers’ motivation was based on worrying about making mistakes had a higher possibility to be overweight or obese. However, it would be interesting to report that an earlier study on elite junior athletes using the same instrument has shown that father-initiated worry-conducive climates were among the factors predicting male athletes’ perfectionistic cognitions [10]. The effort for perfection and for not disappointing a demanding father is expected to impose increased psychological pressure and to be accompanied by distress that may have implications on their eating habits and consequently on their weight. Moreover, this assumption seems to be in line with a previous finding reporting that father's perfectionism can be a risk factor of eating disorders in adolescents [30]. Both behavioral and biological pathways have been previously suggested in connecting chronic stress and obesity in adults and children, describing emotional “comfort” or impulsive eating and selection of specific foods as behaviors observed in stressed individuals [31]. As far as parents’ authority style was concerned, although statistical significant differences were revealed between boys and girls in parents’ authority style, no association was found with the adolescents’ BMI. It has been previously suggested that adolescents with more authoritarian parents had greater increases in BMI as they transitioned to young adulthood [11]. However, since there is a scarcity of data on this area, the relationship between parental style and perceived parent initiated motivational climate should be further explored in future studies, probably using qualitative research methodology.

The main strength of this study is that it is the one of the few internationally [11,30], and the first in Greece, that has attempted to investigate the role of perceived motivational climate of parents and parental style as factors associated with adolescents’ BMI. Another advantage is that body height and weight were measured instead of using self-reported data, providing accurate evidence about the adolescents BMI. However, some limitations may affect the interpretation of our findings. First of all, the low response rate of students and the even lower of parents, despite the initial moderate response to the completion of the informed consents could have resulted in some selection bias, with probable impact on the prevalence of overweight and obesity in both parents and adolescents. The response rate could have been partly explained by the fact that the researchers were allowed to communicate with parents only indirectly in writing via the school and the students, so they were depended on the efforts of teachers with many competing priorities and the complexities of school operations and scheduling. The same limitations have been observed in previous research [32]. However, since parents or adolescents with a higher BMI could have an increased possibility to avoid participating, we believe that this limitation could have underestimated the already high prevalence of overweight/obesity reported in parents and could reflect an under estimation of the prevalence of overweight and obesity in both parents and students. Another limitation of the study could be that some information bias may have occurred given that the assessments of anthropometric variables were based on self reports. There are some evidence indicating that BMI values calculated from self-reports could differ compared to those calculated from measured values- being usually lower- with women having a higher possibility to be under-reporters, whereas men could be over-reporters [33]. However, anthropometric measurements of parents have been self-reported in the majority of previous studies, while possible underestimation of maternal BMI could reinforce our findings, since the prevalence of overweight and obese mothers was high.

## 5. Conclusions

The present study highlights the importance of overweight and obesity in adolescents as a public health problem in this population in central Greece including the mother’s educational level and the type of worried father among the factors associated with obesity in adolescents. Our findings emphasized the central role that mothers are expected to play providing the appropriate role model in their children and in implementing a more healthy diet and lifestyle for their family. The identified factors should be taken into consideration in the development of obesity prevention strategies by policy-makers, mass-media, health professionals and teachers targeting more effectively on both children’s and their parents’ needs.
